# Growth behavior and mRNA expression profiling during growth of IPEC-J2 cells

**DOI:** 10.1186/s13104-024-06812-w

**Published:** 2024-06-05

**Authors:** A. Ronja D. Binder, Veronika Mussack, Benedikt Kirchner, Michael W. Pfaffl

**Affiliations:** Division of Animal Physiology and Immunology, TUM School of Life Sciences, Technical University of Munich, Weihenstephaner Berg 3, 85354 Freising, Germany

**Keywords:** mRNA, ECIS, Next generation sequencing (NGS), IPEC-J2, Growth behavior

## Abstract

**Objective:**

The IPEC-J2 cell line is used as an in vitro small intestine model for swine, but it is also used as a model for the human intestine, presenting a relatively unique setting. By combining electric cell-substrate impedance sensing, with next-generation-sequencing technology, we showed that mRNA gene expression profiles and related pathways can depend on the growth phase of IPEC-J2 cells. Our investigative approach welcomes scientists to reproduce or modify our protocols and endorses putting their gene expression data in the context of the respective growth phase of the cells.

**Results:**

Three time points are presented: (TP1) 1 h after medium change (= 6 h after seeding of cells), (TP2) the time point of the first derivative maximum of the cell growth curve, and a third point at the beginning of the plateau phase (TP3). Significantly outstanding at TP1 compared to TP2 was upregulated PLEKHN1, further FOSB and DEGS2 were significantly downregulated at TP2 compared to TP3. Any provided data can be used to improve next-generation experiments with IPEC-J2 cells.

**Supplementary Information:**

The online version contains supplementary material available at 10.1186/s13104-024-06812-w.

## Introduction

The cell line IPEC-J2 (RRID: CVCL_2246) represents a unique tool for in vitro research with small-intestine models. As one of the few cell models for the small intestine [1], they fill a niche. In particular, this is in relation to the fact that this spontaneously infinite cell line is non-tumorous and non (plasmid) transformed but spontaneously immortalized [2, 3]. IPEC-J2 cells are widely used for infection experiments with swine-specific pathogens [1, 4, 5], but also with non-swine-specific pathogens [6, 7]. Further, non-pathogen (food additives and inflammation) experiments were carried out [8–11]. Results may partially be used as a model for the human small intestine [3, 12]. Since IPEC-J2 cells are not derived from rodents but from unsuckled piglets, they are relatively comparable to human cell models [2, 3]. The similarity between swine and humans can also be observed at a superior level, as results of gene expression profiles with this cell line can be transferred to human models [13].

One frequently used method for assessment of effects of substances, pathogens, or similar is next-generation sequencing (NGS). It can be used for gene expression profiles or evaluating influenced pathways. For this purpose, mRNAs, for example, can be extracted and analyzed. The behavior of epithelia-forming cells can vary during different growth phases or after epithelial closure during epithelial polarization [14]. Pi et al., for example, showed that after cell differentiating of IPEC-J2 cells gene expression profile changed [15], but not much is known about the time during epithelial closure. It has to be noted that growing cell communities are also changing their gene expression profile in dependency on their respective growth phase [16]. This factor may influence the output of experiments during or after epithelial closure. Also, for experiments with IPEC-J2 cells, gene expression profiles may be used increasingly often [9, 11, 17] and thus represent an indispensable tool for future studies.

As the respective growth phase of the cells can influence experiments [16], it may often be required to pay attention to the actual growth phase during design and conduction of a new, NGS-based study. Harmonizing the growth phase in similar studies based on NGS experiments might help to analyze data. A strong and growth-dependent influence on the gene expression profile at specific growth phases may support or cover treatment effects and influence experimental outcomes.

Our study aimed to provide comparative data on the gene expression profile of IPEC-J2 cells based on mRNA sequences at different time points of the growth curve at a given seeding density and a “treatment” protocol (change of the medium). The cells’ growth curve (epithelial closure plotted against the time) was determined via electric cell-substrate impedance sensing technology (ECIS) for a specific seeding concentration. To enhance comparability of further mRNA NGS studies with this cell line, cell counting numbers at the respective sampling-time points as well as the expected RNA output were also given.

## Materials and methods

### Cell culture conditions and handling

For all experiments, IPEC-J2 cells were used in passages 1–5 after thawing and they were cultured in Dulbecco’s Modified Eagle Medium/F-12 Nutrient Mixture (Ham), [+] L-Glutamine (DMEM/F12, Gibco, Schwerte, Germany) including 5% fetal calf serum (FCS, Sigma, Hamburg, Germany) and 100 Units/ml Penicillin/Streptomycin (Sigma, Hamburg, Germany). Detaching of cells was performed with pre-warmed trypsin (0.25% Trypsin/0.02% EDTA, Sigma, Hamburg, Germany). See supplementary for more details (Text S1).

IPEC J2 cells were monitored for *Mycoplasma sp.* via PCR (Thermo Fisher Scientific, Schwerte, Germany) and DAPI staining (mycoplasma test kit A3744, AppliChem, Darmstadt, Germany).

For both experiments (ECIS and NGS), the cells were seeded in a concentration of 12,500 cells per cm² growth area, adapted to *Binder and Spiess et al.* [18]. Afterwards, the cells were allowed to settle for 5 h, and the medium was replaced with fresh, pre-warmed medium. This step was included to simulate a possible treatment in the experimental design.

### Electric Cell-substrate Impedance Sensing (ECIS) experiment

ECIS [19] was used to assess sampling time points. Before cell seeding, ECIS wells were incubated with cell culture medium until a stable baseline was achieved. The baseline was measured for a minimum of 1 h to display a stable background. In total, 400 growth curves were assessed. Processing of the growth curves in R and information about repetitions can be found in the supplementary material (Text S2).

### Next-generation sequencing (NGS) experiment and cell count

For the NGS experiment, cells were seeded in 12-well plates (Grainer, cellstar, Bio-one GmbH, Austria). In each 12-well plate, three of the lateral wells were used to ensure that each repetition was performed equally. For each of the three sampled time points (TP1-TP3), an independent plate was used so that changes in the microenvironment during cell growth were avoided. Still, all time points were seeded with the same cell suspension simultaneously. After reaching the respective time point (TP1-TP3), development of the cells was stopped by washing the cells with 2 ml pre-warmed DPBS and a treatment with respectively 700 µl QIAzol (QIAzol lysis reagent, Qiagen, Venlo, Netherlands, 5 min, two of the three wells of each time point) or pre-warmed trypsin (one of the three wells of each time point until cells detached.

Trypsin-treated cells were removed from the wells and counted (Fig. S1). Detailed processing of the NGS experiment and employed kits can be found in the supplementary material (Text S3). The experiment was repeated three times on different days with different cell suspensions.

### Statistics and software: general information, data processing, and pathway analysis

Significance was accepted for *p*-values ≤ 0.05. All statistical evaluations were performed with R software [20], and growth curves were assessed according to an algorithm developed by Binder and Spiess et al. [18]. Mean and standard derivation (± SD) are given when applicable. Further, for NGS data analysis, adapter sequences were trimmed with Cutadapt [21], and the raw sequencing data were quality-controlled with FastQC [22]. STAR [23] and RSEM [24] were used for alignment and annotation. Differential gene expression was analyzed with the identified transcriptomic profiles with DESeq2 [25], and significant changes were filtered by |log2 fold changes| ≥ 1 a mean normalized expression profile of 50 reads over all samples and adjusted p-value ≤ 0.05.

Pathways of the expressed mRNAs were analyzed with Reactome (Table S1) [26]. Therefore, TP1 was compared to TP2, and TP2 to TP3, respectively.

## Results

### Extracted time points of the ECIS experiment

The following three time points were extracted from the ECIS experiment: (TP1) 6 h after seeding (i.e. 1 h after treatment); (TP2) the time point in the log phase, when the first derivative maximum (FDM) of smoothing spline curve in hours after seeding, was reached (35.71 h ± 5.58 h, TP2) and the third point in the growth phase, (TP3) when all cells had reached the plateau, seg3 (segmented regression curve) in hours after seeding (45.34 h ± 8.67 h).

Based on the ECIS results, sampling for NGS experiments was uniformly set to the following: TP1 = 6 h, TP2 = 36 h and TP3 = 60 h (TP3 is including a safety factor of approximate 1.75 x standard derivation).

### Expectable quantity and quality of the harvested cells/RNA

The cell count in relation to the RNA concentration [ng/µl] showed a correlation of R²=0.87, respectively, of an adjusted R² of 0.85. Dependence can be represented by the following equation: y = 0.002*x 41.654.

### Analysis of NGS

**Fig. 1:**
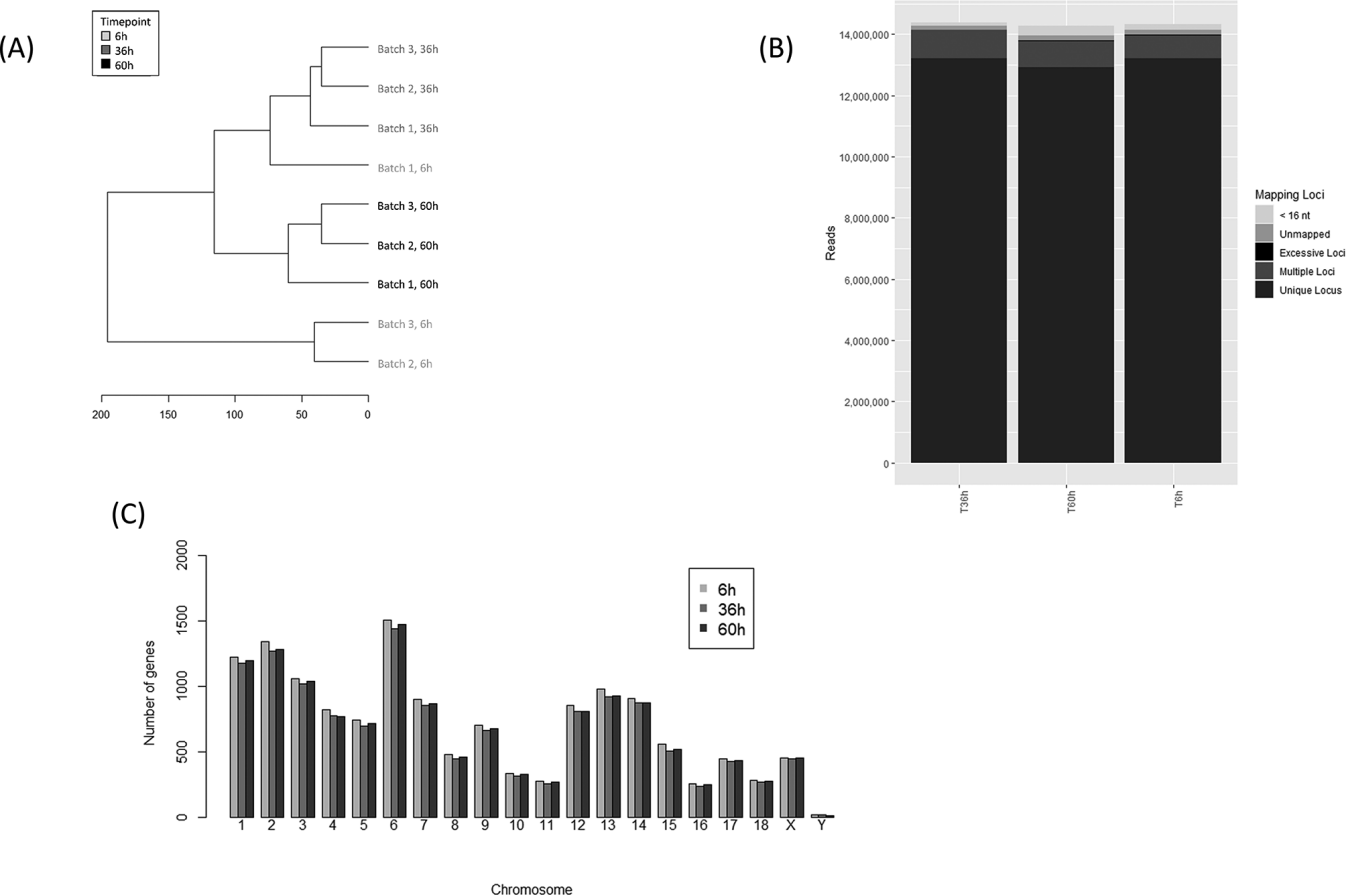
Characterization of batches (**A**) and reads per loci (**B**) at three time-points and (**C**) distribution of significantly regulated genes per chromosome

Cluster analysis showed apparent differences in the mRNA expression between the different growth phases (Fig. 1A). A consideration of the reads per locus showed that, by far, the majority of reads were characterized as unique loci at all time-points (Fig. 1B). The number of significantly regulated genes per chromosome was assed. They showed an even distribution pattern per chromosome over the different time-points (Fig. 1C).

**Fig. 2:**
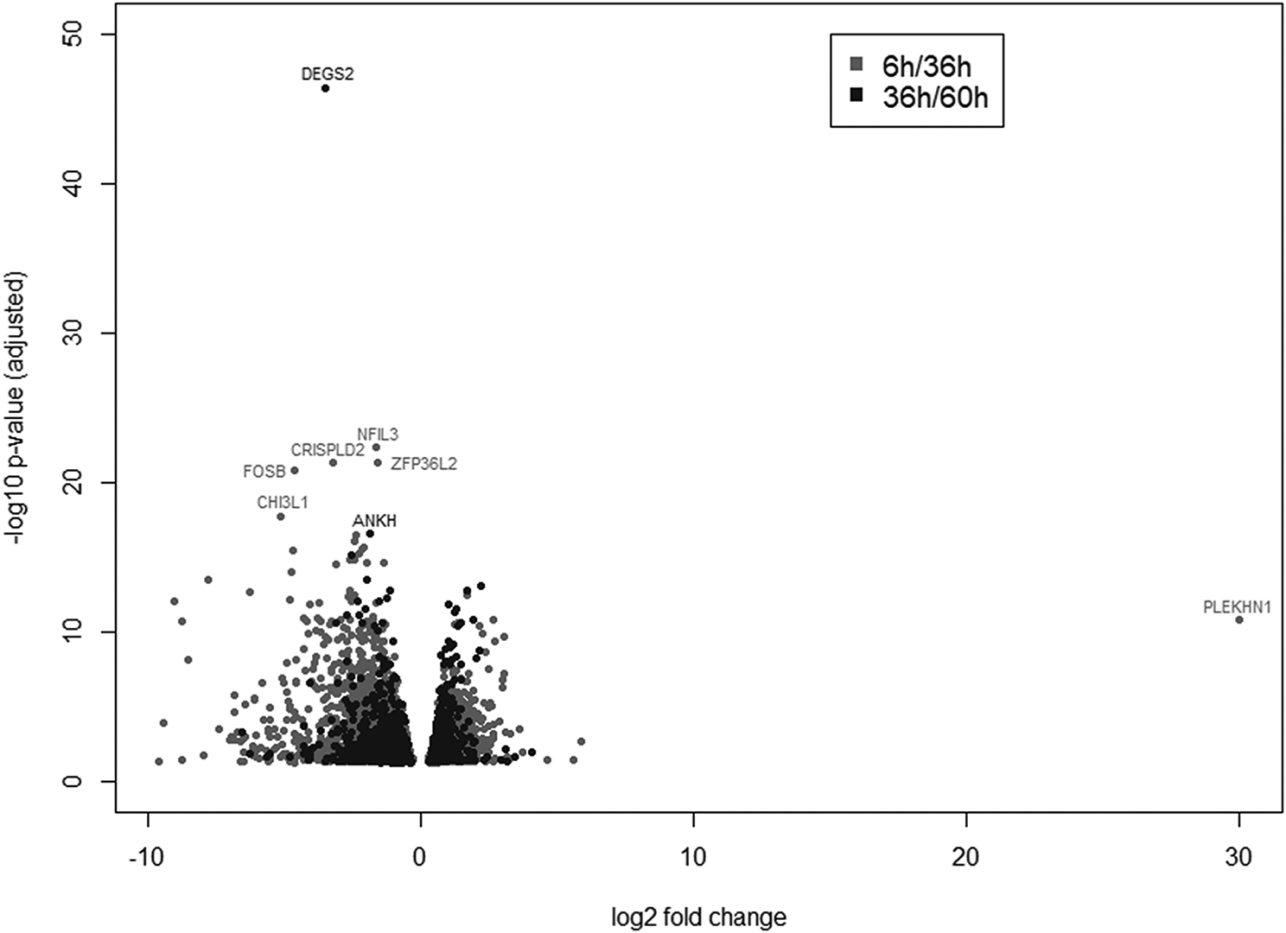
Differentially expressed genes at the chosen time-points. Dots represent log2 fold changes and its respective adjusted -log10 p-value between time-point 36 h compared to 6 h (light grey) and between time-point 60 h compared to 36 h (dark grey)

Differentially expressed genes showed a stronger distributed pattern in relation to log2 fold change and the respective adjusted –log 10 p-value for time-point 36 h compared to 6 h as for time-point 60 h compared to 36 h (Fig. 2). Particularly outstanding genes at time-point 60 h compared to 36 h were DEGS2 (Delta 4-Desaturase, Sphingolipid 2) and FOSB (FosB Proto-Oncogene, AP-1 Transcription Factor Subunit) (both downregulation, Fig. 2), and at time-point 36 h compared to 6 h, PLEKHN1 (pleckstrin homology domain containing N1, upregulation) with a log2 fold change of over 30 (Fig. 2). A comprehensive list of all differentially expressed mRNAs during cell growth is given in Additional file 2. Raw sequencing data are provided in the European Nucleotide Archive (ENA accession ID: PRJEB74363).

### Pathway analysis via reactome

Analysis of significantly expressed genes via Reactome showed regulations, particularly in the cell cycle and rRNA processing pathways. Cell cycle activities were in focus during logarithmic cell growth (TP1/TP2). After reaching the plateau phase (TP2/TP3), rRNA processing and modification activities took up the largest part. The 10 most relevant pathways detected by Reactome can be found in the supplementary material (Table S1).

## Discussion

Sampling of different time points and comparison to a non-treated control group can improve findings of treated cells, as we showed previously [27]. Derived from the corresponding ECIS growth curve, three possible time points were evaluated and set appropriately, at which a sampling of IPEC-J2 cells can be recommended if seeding and treatment are performed as described in our protocol.

It has to be kept in mind that a fully polarized monolayer of IPEC-J2 cells was most likely not formed for the chosen time points, as this was not the focus of our study. A polarized monolayer might be reached later within 1–2 weeks, depending on cell culture medium, according to Vergauwen [3].

As expected, assessing gene expression profiles of IPEC-J2 cells at different phases during cell growth resulted in different findings, due to different growth phases of cells (Fig. 2).

Significantly outstanding at TP1 compared to TP2 was upregulated PLEKHN1 (Fig. 2) that is, among others involved in phospholipid binding [28] and apoptotic processes [29]. Kuriyama et al. showed that expression of PLEKHN1 was induced by stress, and it might be an indicator of damaged cells. As a consequence, they were able to observe an increased in vivo survival rate of cancer cells in PLEKHN1 knock-out mice [29]. This indicates the logarithmic growth phase of small intestine cells as a suitable sampling time point in gene expression studies regarding the behavior of damaged cells, among others, in correlation with intestinal cancer.

For mice, it is known that small intestine stem cells can show alterations during aging [30]. Nefzger et al. found a correlation between the aging of intestinal stem cells and three key transcription factors, including the down-regulation of FOSB in aged cells [31]. In our study, FOSB was significantly downregulated at TP2 compared to TP3 (Fig. 2). This indicates a similar behavior of FOSB during cell growth of small intestine cells as during the aging of intestine stem cells. This gene might, therefore, also be suitable as an indicator of a late growth phase of the cell growth curve of small intestine cells. This finding also supports FOSB for clinical applications regarding regeneration of the small intestine, as Nefzger et al. propose [31].

The same growth phase might be of interest for studies concerning DEGS2. DEGS2 is encoding Delta(4)-desaturase sphingolipid 2 and is therefore involved in the sphingolipid biosynthesis [32] and regulation of phytoceramide and dihydroceramide in the intestine [33]. In our study, DEGS2 was significantly downregulated at TP2 compared to TP3 (Fig. 2). Dysregulation of DEGS2 might be associated with multiple diseases, such as cancer (Guo et al., 2021) or inflammation-associated diseases [34, 35]. Therefore, this specific growth phase might be specifically sensitive as a model for those diseases.

Figure S1 can be consulted as a first estimation of how many cells have to be harvested to achieve the desired concentration of RNA. Strongly deviating concentrations between replicates may indicate varying seeding concentrations, cell quality, or handling or incubation conditions (e.g. temperature changes, handling time). It has to be assumed that, in particular between different cell dilutions, seeded at different days with maybe different passage times, it may often not be perfectly possible to synchronize the growth behavior of the cells (compare Fig. 1A).

## Conclusion

Our experiments showed that the respective sampling time point during cell growth can influence the outcome of a mRNA gene expression experiment with an IPEC-J2 cell model. Accordingly, this aspect should be considered when working with cells as an in vitro model. Therefore, unequivocally, naming the sampling time point (growth phase of cell line) might improve the comparability of different studies. Further, choosing the appropriate sampling time point or the subsequent sampling of multiple time points during cell growth might improve outcomes.

## Limitations

It has to be kept in mind that different cell lines might vary differently in the intensity of how the gene expression profiles differ between multiple time points during cell growth.

### Electronic supplementary material

Below is the link to the electronic supplementary material.

Supplementary Material 1

Supplementary Material 2

## Data Availability

Additional data can be found in Additional file 1 and Additional file 2. Further, raw sequencing data can be found in the European Nucleotide Archive (ENA, accession ID: PRJEB74363).

## Abbreviations

TP1, TP2, TP3: Time point 1-3
NGS: Next generation sequencing
mRNA: Messenger ribonucleotide acid
ECIS: Electric Cell-substrate Impedance Sensing
DMEM/F12: Electric Cell-substrate Impedance Sensing
FDM: First derivative maximum
Seg: Segmented regression
ENA: European Nucleotide Archive
