# Supplementary material for: Growth behavior and mRNA expression profiling during growth of IPEC-J2 cells
Source: BMC Res Notes. 2024 Jun 5;17:154. doi: 10.1186/s13104-024-06812-w (PMC11155027; doi:10.1186/s13104-024-06812-w)
Supplement: Supplementary file 1 — Supplementary Material 1 [file 13104_2024_6812_MOESM1_ESM.pdf]

Additional file 1

Growth behavior and mRNA expression profiling during growth of IPEC-J2 cells

A. Ronja D. Binder[1*], Veronika Mussack[1], Benedikt Kirchner[1], Michael W. Pfaffl[1]

1) Division of Animal Physiology and Immunology, TUM School of Life Sciences, Technical University of Munich, Weihenstephaner Berg 3, 85354 Freising, Germany

* Corresponding author: Ronja.Binder@tum.de

Text S1 Cell culture conditions and handling of IPEC-J2 cells

Text S2 Electric cell-substrate sensing experiment (ECIS)

Text S3 Next-generation sequencing experiment (NGS) and cell count

Table S1 The 10 most relevant pathways detected by Reactome

Fig. S1 RNA concentration [ng/µl] in relation to counted cells

Text S1 Cell culture conditions and handling of IPEC-J2 cells

IPEC-J2 cells were incubated with 5% Co_2 and ~90% air humidity (Heracell VIOS160i, Thermo fisher scientific, Schwerte, Germany). Cells were cultured in cell culture dishes (diameter 35mm, 60mm, or 100mm, Greiner Bio-One, Kremsmünster, Germany) with Dulbecco's Modified Eagle Medium/F-12 Nutrient Mixture (Ham), [+] L-Glutamine (DMEM/F12, Gibco, Schwerte, Germany) including 5% fetal calf serum (FCS, Sigma, Hamburg, Germany) and 100 Units/ml Penicillin/Streptomycin (Sigma, Hamburg, Germany). Experiments were performed in 12-well cell culture dishes (Grainer, cellstar, Bio-one GmbH, Austria) and in 10E ECIS cell culture dishes (8W10E, ibidi, Gräfelfing, Germany). For any experiments and splitting, the cells were washed two times with pre-warmed Dulbecco's Phosphate Buffered Saline (DPBS, Sigma, Hamburg, Germany) and detached with pre-warmed trypsin (0.25% Trypsin/0.02% EDTA, Sigma, Hamburg, Germany). After detaching of the cells, trypsin activity was blocked with culturing medium, and cells were counted if necessary. Cells were pelleted (pelleting: centrifugation: 300 g RCF, 5 min), the medium was removed, and cells were diluted in the desired concentration and seeded. Cell counting was performed with a Neubauer-improved cell counting chamber. If a container or liquid came in contact with cells, the opening was conducted under a workflow bench, except for the NGS experiment, as RNA was extracted, respectively cells were counted but not further used.

Text S2 Electric cell-substrate sensing experiment (ECIS)

The timepoint of seeding of the cells was set to value zero hours, the baseline of all growth curves was shortened to 1-10 hours (depending on the measured baseline time) and the plateauphase was shortened to 70 hours after timepoint zero. Growth curves of all monitored wells were uploaded [1] and processed [2] in R, and the baseline was extended to -50 hours. Afterwards, the fitting of the curves was performed.

The experiment was repeated ten times on different days and with different suspensions of cells with two wells and two ECIS dishes for each repetition (n = 10*2*2 = 400) so that 400 growth curves were assessed in total. During each repetition, four wells were used as a negative control (only medium change, but no seeded cells) so that biological contaminations were excluded as far as possible.

Text S3 Next-generation sequencing experiment (NGS) and cell count

The QIAzol-treated cells were washed off and transferred to DNA LoBind tubes (Eppendorf, Hamburg, Germany). DNA LoBind tubes were vortexed roughly for 10 seconds and put directly on ice. Afterwards, they were stored immediately at -80°C until further processing.
For all timepoints (TP1-TP3), one of the two QIAzol-treated wells was used for total RNA extraction with the Qiagen miRNeasy Mini Kit (Qiagen, miRNeasy Mini Kit, Hilden, Germany). Further, RNA quantity and quality were assessed with the Bioanalyzer (Bioanalyzer 2100, Agilent, USA, Agilent RNA-6000 Nano Kit, Frankfurt, Germany, Fig. S1). For further processing, the mRNA was enriched with a magnetic isolation module kit (NEBNext Poly (A) mRNA, 24 reactions, NewEngland BioLabs, Frankfurt, Germany). The library preparation was performed with the NEBNext Ultra II RNA Library Prep kit (with Sample Purification Beads, 24 reactions, NewEngland

BioLabs, Frankfurt, Germany) and the NEBNext Multiplex Oligos for Illumina kit (Dual Index Primers Set 1 NewEngland Biolabs, Frankfurt, Germany). NovaSeq 6000 S1 Reagent kit (100 cycles, 1x 100 bp, Illumina, New York, US) was performed prior to the NGS step.

References

1. R Core Team. R: A language and environment for statistical computing. R Foundation for Statistical Computing, Vienna, Austria. 2020. https://cran.r-project.org/.

2. Binder ARD, Spiess A-N, Pfaffl MW. Modelling and Differential Quantification of Electric Cell-Substrate Impedance Sensing Growth Curves. Sensors. 2021;21:5286.

Table S1: The 10 most relevant pathways detected by Reactome for comparison of time-point 6h vs. 36h (TP1/TP2), respectively 36h vs. 60h (TP2/TP3)

Compared time-point	Pathway name	Entities (found)	Reactions (found)
**TP1/TP2**	Cell cycle	292/734	433/451
**TP1/TP2**	Cell cycle, mitotic	244/596	338/352
**TP1/TP2**	Cell cycle, checkpoints	118/279	54/56
**TP1/TP2**	TP53 Regulates Transcription of Genes Involved in G1 Cell Cycle Arrest	17/20	17/17
**TP1/TP2**	Mitotic Spindle Checkpoint	55/111	7/7
**TP1/TP2**	DNA strand elongation	25/38	14/15
**TP1/TP2**	Diseases of mitotic cell cycle	25/38	4/5
**TP1/TP2**	Aberrant regulation of mitotic cell cycle due to RB1 defects	24/36	2/3
**TP1/TP2**	rRNA modification in the nucleus and cytosol	39/72	7/8
**TP1/TP2**	Activation of ATR in response to replication stress	25/39	9/9
**TP2/TP3**	rRNA modification in the nucleus and cytosol	32/72	5/8
**TP2/TP3**	rRNA processing in the nucleus and cytosol	51/208	12/15
**TP2/TP3**	Major pathway of rRNA processing in the nucleolus and cytosol	47/189	7/7
**TP2/TP3**	rRNA processing	53/246	14/21
**TP2/TP3**	GRB7 events in ERBB2 signaling	5/6	1/1
**TP2/TP3**	FOXO-mediated transcription of cell cycle genes	10/27	18/22
**TP2/TP3**	Metabolism of RNA	116/829	106/189
**TP2/TP3**	RNA Polymerase III Transcription Termination	8/23	2/2
**TP2/TP3**	FOXO-mediated transcription of cell death genes	8/23	14/15
**TP2/TP3**	ISG15 antiviral mechanism	18/83	6/16

P-value for all pathways (found entities) was <0.01

Fig. S1 RNA concentration [ng/µl] in relation to the counted cells

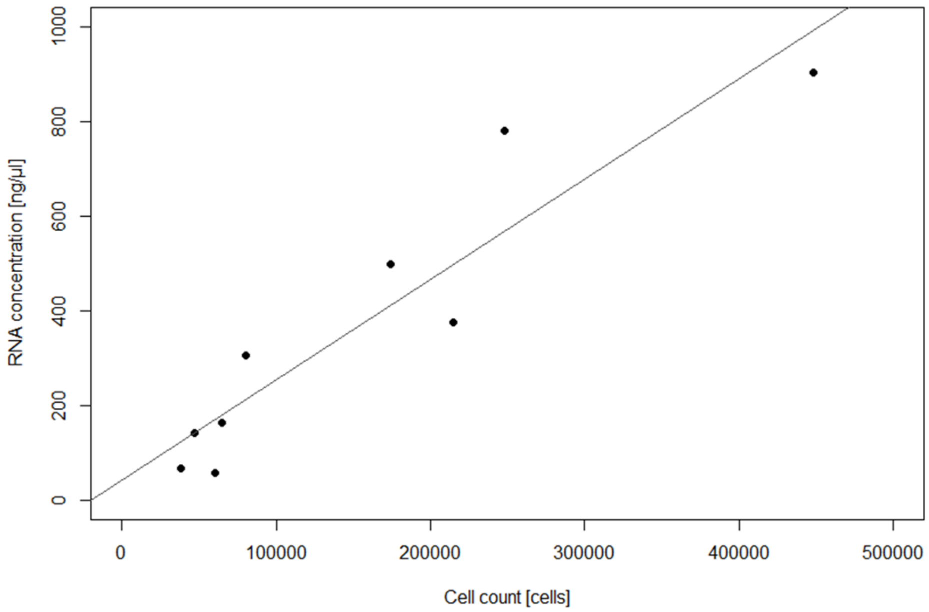

R^2: 0.87, adjusted R^2: 0.85.
